# Cloning, expression and seroreactivity of the recombinant lipopolysaccharide assembly protein - D (LptD) from *Bartonella bacilliformis*

**DOI:** 10.17843/rpmesp.2022.391.9292

**Published:** 2022-03-31

**Authors:** Astrid Flores-Nuñez, Gladis Ventura, Henri Bailon, Adolfo Marcelo, Gustavo Sandoval, Carlos Padilla-Rojas

**Affiliations:** 1 Laboratorio de Biotecnología y Biología Molecular, Centro Nacional de Salud Pública, Instituto Nacional de Salud, Lima, Peru. Laboratorio de Biotecnología y Biología Molecular Centro Nacional de Salud Pública Instituto Nacional de Salud Lima Peru; 2 Grupo de Investigación en Bioinformática y Biología Estructural, Universidad Nacional Mayor de San Marcos, Lima, Peru. Universidad Nacional Mayor de San Marcos Grupo de Investigación en Bioinformática y Biología Estructural Universidad Nacional Mayor de San Marcos Lima Peru; 3 Laboratorio de Referencia Nacional de Metaxénicas Virales, Centro Nacional de Salud Pública, Instituto Nacional de Salud, Lima, Peru. Laboratorio de Referencia Nacional de Metaxénicas Virales Centro Nacional de Salud Pública Instituto Nacional de Salud Lima Peru

**Keywords:** *Bartonella* infections, LptD protein, *Bartonella bacilliformis*, Recombinant proteins, Vaccine Antigenicity, Computational biology

## Abstract

**Objective.:**

To evaluate in silico and at the serological level the antigenic potential of the recombinant extracellular domain of the lipopolysaccharide assembly protein - D (LptD) of Bartonella bacilliformis (dexr_LptD).

**Materials and Methods.:**

Through in silico analysis, we selected a B. bacilliformis protein with antigenic and immunogenic potential. The selected protein gene was cloned into Escherichia coli TOP10 and expressed in Escherichia coli BL21 (DE3) pLysS. Recombinant protein was expressed using isopropyl-β-D-1-thiogalactopyranoside (IPTG) and induction conditions were optimized. Finally, it was purified with Ni-IDA resin (His60 Ni Superflow) and a Western Blot assay was conducted.

**Results.:**

In silico, the selected protein was LptD because it is located in the outer membrane and is antigenic and immunogenic. Optimized conditions for dexr_LptD induction were 0.5 mM IPTG, 16 hours, TB (Terrific Broth) medium, 3% (v/v) ethanol, 28 ºC, OD600: 1-1.5 and 200 rpm. Purification was carried out under denaturating conditions on a small scale and we obtained 2.6 μg/mL of partially purified dexr_LptD. The Western Blot assay showed a positive reaction between the sera from patients with Carrión's Disease and dexr_LptD, which shows the antigenicity of dexr_LptD.

**Conclusions.:**

The dexr_LptD shows antigenicity both in silico and at the serological level, these results are the basis for further studies on vaccine candidates against Carrion’s Disease.

## INTRODUCTION

*Bartonella bacilliformis* is the etiological agent of Carrion’s disease (CD), whose vector is the sandfly of the genus *Lutzomyia spp*. CD is a neglected disease in Peru, Colombia and Ecuador, and during its first development phase it has high lethality and mortality rates (40 - 88%) in patients without antimicrobial treatment ^1^. Research on CD has been aimed at more sensitive detection techniques, such as the polymerase chain reaction (PCR) ^2^; the identification of new antigenic and immunogenic proteins to be used in serological diagnosis ^3^ ^-^ ^8^ and very few to the the search for vaccine candidates ^9^ ^,^ ^10^, therefore, currently there is no vaccine for this disease.

Reverse vaccinology, a methodology used in this study, allows the identification of antigens and immunogens without the need to cultivate the pathogen; this is achieved through the use of bioinformatics programs, a step prior to experimental trials ^11^. This methodology allowed synthesizing a multiepitope protein of *B. bacilliformis*, which was able to induce an immune response in mice, which positions it as a good vaccine candidate against CD ^9^.

Outer membrane proteins are used within the proteome of a microorganism to search for vaccine candidates ^12^ ^,^ ^13^, due to their ability to interact with the host immune system. Thus, we have the lipopolysaccharide (LPS) transport system LptABCDEFG, a protein complex responsible for the translocation of LPS from the inner membrane to the outer membrane ^14^ ^-^ ^16^. The lipopolysaccharide assembly protein - D (LptD), part of the LptABCDEFG system, has been found to be highly immunogenic, therefore, a good vaccine candidate for other microorganisms such as *Vibrio parahaemolyticus*; in addition, it could be used in immunotherapy for being a key piece in the final process of LPS translocation ^17^ ^,^ ^18^.

Therefore, this research aims to evaluate *in silico* and at the serological level the antigenic potential of the recombinant extracellular domain of the lipopolysaccharide assembly protein - D (LptD) of *Bartonella bacilliformis* (dexr_LptD). Our results will be important to the basic line of research for the development of a vaccine against CD.

**KEY MESSAGES**

Motivation for the study: Currently there is no vaccine against Carrion’s disease, which affects vulnerable populations. On the other hand, we have reverse vaccinology as a tool that helps us in the selection of vaccine candidates.

Main findings: The recombinant extracellular domain of the lipopolysaccharide assembly protein - D (LptD) of *B. bacilliformis* showed antigenicity *in silico* and at serological level.

Implications: The results contribute to the research line on vaccine candidates against Carrion’s disease.

## MATERIALS AND METHODS

**Figure 1:**
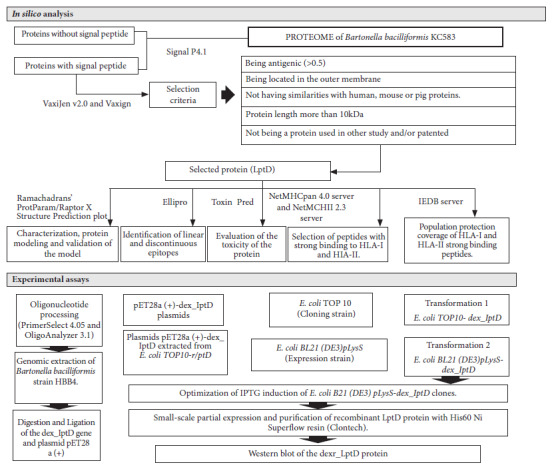
Study flowchart. *In silico* analysis: shows the bioinformatics programs and criteria for the selection of the studiedprotein. Experimental assays: sequence of procedures performed to obtain and evaluate the recombinant extracellular domain of the *Bartonella bacilliformis* lipopolysaccharide assembly protein - D (dexr_LptD). LptD (lipopolysaccharide assembly protein - D), HLA-I and HLA-II (human leukocyte antigens I and II), IPTG (isopropyl-β-D-thiogalactopyranoside). Bioinformatics programs: SignalP4, VaxiJen v2.0 and Vaxign, ProtParam/ Raptor X Structure Prediction/ Ramachandran plot, Ellipro, Toxin Pred, NetMHCpan 4.0 Server and NetMHCII 2.3 Server, IEDB Server, PrimerSelect 4.05 and OligoAnalyzer 3.1.

The methodology included two stages: *in silico* analysis and experimental trials (Figure 1).

### *In silico* analysis

We selected an outer membrane protein with antigenic and immunogenic potential from the genome and proteome of *B. bacilliformis* KC583 (ID Assembly: GCA_000015445.1). The SignalP4.1 program (https://services.healthtech.dtu.dk/service.php?SignalP-4.1) was the first filter, which allowed the selection of proteins with signal peptide i.e., proteins located in the cell membrane. Signal P4.1 is one of the best signal peptide predictors ^19^. The following filters were applied with the programs VaxiJen v2.0 (http://www.ddg-pharmfac.net/vaxijen/VaxiJen/VaxiJen.html) and Vaxign (http://www.violinet.org/vaxign/), which allowed selection of proteins located in the outer membrane with antigenic potential and identification of similarity to human, mouse or pig proteins, as well as estimation of protein length and probability of being adhesins. VaxiJen v2.0 and Vaxign are programs with fast graphical interface and intuitive use, which are updated regularly and have been the most cited in comparison with Nerve, Jenner-predict, Bowman-Heinson and VacSol programs ^20^ ^,^ ^21^.

The physicochemical properties (molecular weight, theoretical pI, amino acid composition, atomic composition, extinction coefficient, estimated half-life, instability index and aliphatic index) of the pre-selected proteins were obtained with ProtParam (https://web.expasy.org/protparam/). The proteins were modeled with RaptorX Property (http://raptorx2.uchicago.edu/StructurePropertyPred/predict/) and validated with the Ramachandran plot, using the program Rampage: Ramachandran Plot Assessment (http://mordred.bioc.cam.ac.uk/~rapper/rampage.php). It should be noted that RaptorX Property is among the top five automated protein modeling programs ^22^. Protein spatial orientation was assessed with the PPM server program (https://opm.phar.umich.edu/ppm_server).

Once the protein of interest was obtained, we used the Ellipro server (http://tools.iedb.org/ellipro/) to identify the presence of linear and discontinuous epitopes. This server selects peptides with a score higher than 0.5 and qualifies them as antigens. On the other hand, Toxin Pred - Protein Scanning (http://crdd.osdd.net/raghava/toxinpred/) evaluated the toxicity of the peptides.

A protein with vaccine potential must possess epitopes capable of interacting with the human leukocyte antigen (HLA) class I and II system. The identification of peptides with strong binding to HLA-I and II was carried out with the NetMHCpan 4.0 Server (https://services.healthtech.dtu.dk/service.php?NetMHCpan-4.1) and NetMHCII 2.3 Server (https://services.healthtech.dtu.dk/service.php?NetMHCII-2.3) programs, respectively.

The IEDB program (https://www.iedb.org/) evaluated the percentage of coverage, in the country, in South America and in the world, of the interaction of the most frequent HLA-I and HLA-II in the Peruvian population with the selected peptides. Annex 1 shows the most frequent and most important HLA-I and HLA-II in the Peruvian population (data obtained by communication with biologist Adolfo Marcelo of the Peruvian National Institute of Health).

## EXPERIMENTAL TRIALS

### Oligonucleotide design

Oligonucleotides were designed using PrimerSelect 4.05 (ADNSTAR Inc. Madison, USA) and OligoAnalyzer 3.1 programs from IDT (www.idtdna.com). The forward and reverse oligonucleotides were modified to include the cut sites for the restriction enzymes NcoI (C/CATGG) and XhoI (C/TCGAG), respectively. A six-thymine tail was added to the 5' end of the oligonucleotides to ensure cutting efficiency. The oligonucleotides were: forward 5΄-TTTTTTCCATGGTACCGGACCCAGCTGAAA-3΄, and reverse 5΄-TTTTTTCTCGAGTAAATTGAGTTTTTTTTATTTTTTTTTTGACCGAAATCT-3΄.

### Amplification of the lptD extracellular domain gene (*dex_lpt*D gene).

Genomic DNA from *B. bacilliformis* strain HBB4 from Huacabamba (Piura, Peru) was used for gene amplification by PCR ^23^. We used the AmpliTaq Gold™ DNA Polymerase with Gold buffer and MgCl_2_ (Applied Biosystems) kit. The concentrations of the PCR buffer mix were: 2.5 mM MgCl_2_, 1X Gold buffer, 1 µM forward oligonucleotides, 1 µM reverse oligonucleotides, 0.2 mM dNTPs and 1 U/reaction of Taq Pol. PCR cycling consisted of an initial denaturation at 95 °C for 10 min, followed by 35 cycles of denaturation at 96 °C for 30 s, hybridization at 58 °C for 30 s and amplification at 72 °C for 2 min; finally, amplification at 72 °C for 10 min.

### Cloning of the *dex_lptD* gene in *E. coli*

The amplified *dex_lptD* gene and the expression plasmid pET28a(+) (Promega Corp.,USA) were digested with the restriction enzymes *NcoI* and *XhoI*, and purified with the PureLink™ Quick Plasmid Miniprep kit (Invitrogen Inc, USA). They were then ligated with T4 ligase (Promega Corp.,USA) and the plasmid pET28a (+) - dex_lptD was formed.

Competent *E. coli TOP10* (cloning strain) and *E. coli Bl21* (*DE3) pLysS* (expression strain) were transformed by the calcium chloride method ^24^. The first transformation was between* E. coli TOP 10 *and plasmid pET28a (+) - *dex_lptD*, which resulted in *E. coli TOP 10 - dex_lptD*, from which plasmids were extracted by the alkaline lysis method ^25^. The extracted plasmids were used to carry out the second transformation to obtained* E. coli BL21(DE3)pLysS - dex_lptD*.

In order to verify the conservation of the *dex_lptD* gene sequence in the transformed clones, they were sequenced according to the method of Sanger *et al*. (1977) and Murray (1989), using the Big Dye Terminator v3.1 Cycle Sequencing kit (AppliedBiosystems), the ABI sequencer, and the Chromas Pro program for their respective analysis.

### Expression and purification of the *dexr_LptD* protein

Recombinant protein expression was optimized by evaluating four parameters: (a) IPTG concentration (Sigma-Aldrich Merck, USA): 0, 0.05, 0.1, 0.25, 0.5, 1, 1. 5, 2 mM IPTG; (b) culture media: LB (Luria Bertani), 2XYT (Yeast Extract Tryptone) and TB (Terrific Broth); (c) induction time: 4, 7, 16 and 24 hours; (d) ethanol concentration: 1%, 2 % and 3% e) induction at different temperatures 20 °C, 28 °C and 37 °C. The culture media were supplemented with kanamycin (20 µg/mL) and chloramphenicol (50 µg/mL).

Two conditions were evaluated for purification: denaturing (8M urea) and non-denaturing. Purification was by affinity with 50 µL of His60 Ni Superflow resin (Clontech); the manufacturer’s recommendations were followed. The Bradford method was used for protein quantification.

### PAGE-SDS electrophoresis and Western Blot detection

PAGE-SDS electrophoresis of the partially purified protein was carried out on a 12% polyacrylamide gel; the gel was photographed and analyzed using QuantityOne 4.6.9 1-D Analysis software.

The western blot assay was performed in a horizontal semi-dry transfer chamber (3 mA/ cm^2^ for 30 min) by using and a diluted mixture of five human sera (0.1%). These sera from the serum library of the Biotechnology and Molecular Biology Laboratory (INS) belonged to people who had CD. For this assay, 345 ng of partially purified recombinant protein was used. Finally, the membrane was photographed and analyzed with QuantityOne 4.6.9 1-D Analysis Software.

## RESULTS

**Figure 2:**
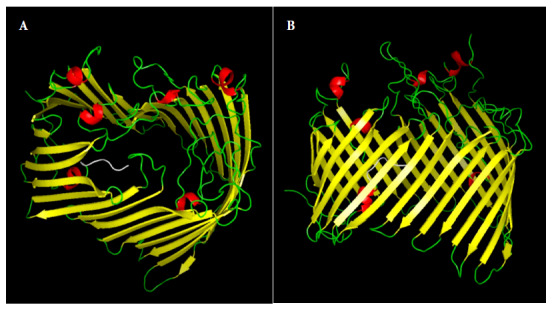
3D model of the recombinant extracellular lipopolysaccharide assembly domain - D of *Bartonella bacilliformis* (dexr_LptD). A: lateral view, B: top view. Secondary structures: beta sheets (yellow), alpha helix (red), loops (green) and histidine tail (white).

The *in silico* analysis of the proteome of *B. bacilliformis* KC583 allowed us to select the LptD protein (ID: A1US68_BARBK) because it met the selection criteria: being antigenic (0.562), located in the outer membrane, no similarity to human, mouse, or pig proteins, and the length of the protein was greater than 10 kDa (86.80 KDa). Subsequent assays were conducted only with the extracellular domain of LptD (67.42 kDa, pI 7.86) because it is the portion of the LptD protein that is located towards the extracellular medium, thus likely to interact with the host immune system. dexr_LptD forms a channel and has asparagine (8.9%) and serine (8.4%) as the most abundant amino acids (Figure 2). The RaptorX program was able to model 100% of the amino acid sequence of the dexr_LptD, using as templates homologous LptD proteins from different species: *E. coli* (PDB: 4RHBA), *Pseudomonas aeruginosa* (PDB: 5IVA), *Yersinia pestis* (5IXMA) and *Shigella flexneri* (PDB: 4Q35A). Likewise, this domain presented 18 linear epitopes and five discontinuous epitopes; in addition, none of the 569 evaluated peptides proved to be toxic. We obtained high population coverage of the dominant HLA class I and class II binding peptides, these were 100.00% for Peru, 93.38% for South America and 99.28% worldwide.

We obtained 116 colonies after the transformation of *E. coli TOP10* strains on the ligation plate and four colonies were obtained on the self-ligating plate, which indicated that most of the transformed bacteria presented plasmid pET28a (+) - *dex_lptD*. Colony PCR was performed on six randomly selected colonies from the ligation plate and all showed amplification products at the expected size (1756 bp). The *lptD* gene from *E. coli TOP10 - dex_lptD* was sequenced and no nucleotide changes were observed with respect to the template (*dex_lptD* gene from *B. bacilliformis KC583*). Once the sequence of the *dex_lptD* gene was confirmed by sequencing, transformation was performed on the *E. coli BL21 (DE3) pLysS* expression strain, and four colonies were randomly selected for colony PCR. PCR results confirmed the correct transformation *into E. coli BL21(DE3)pLysS*.

**Figure 3:**
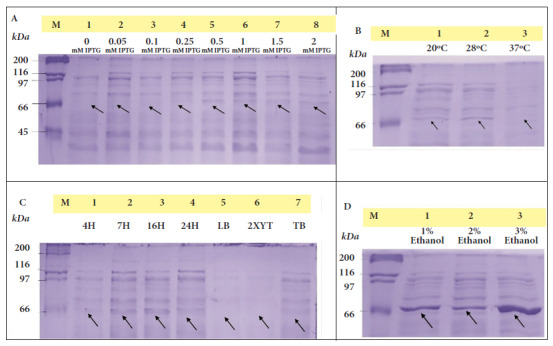
Expression optimization process of the recombinant extracellular lipopolysaccharide assembly domain - D of *Bartonella bacilliformis* (dexr_LptD), where M (marker: Unstained Protein Standards) and arrows indicate the height of the dexr_LptD (67.42 kDa) (induced band).

**Figure 4:**
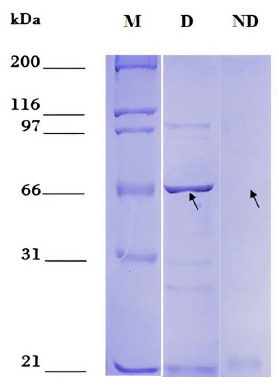
Partial purification of the recombinant extracellular domain of the lipopolysaccharide assembly protein - D of *Bartonella bacilliformis* (dexr_LptD) under denaturing and non-denaturing conditions. M: Marker: Unstained Protein Standards. D: partial purification under denaturing conditions. ND: partial purification under non-denaturing conditions. Arrows indicate the height of dexr_LptD (67.42 kDa).

The optimal induction conditions for dexr_LptD were: 0.5 mM IPTG, TB medium, 16 hours, 3% ethanol, 28 °C and 200 rpm. The optimized purification of dexr_LptD had two lysis steps (the first non-denaturing and the second denaturing), since, when performed only with denaturing buffer, a large amount of unspecific bands were obtained during electrophoresis (Figure 3). The importance of the first lysis step lies in the elimination of the native proteins existing in the sample that interacted with the purification resin. It should be noted that protein purification was not achieved under non-denaturing conditions (Figure 4). The amount of partially purified protein under optimal conditions quantified by the Bradford method was 2.6 µg/mL of dexr_LptD.

**Figure 5:**
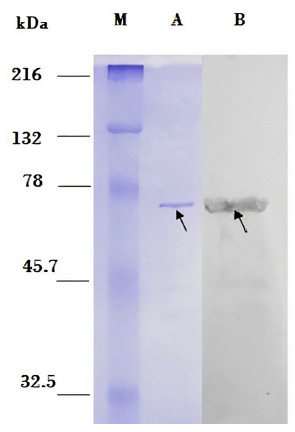
Western Blot. M: Marker: Prestained Natural SDS-PAGE Protein Standards. Left side: PAGE-SDS gel. A: 115 ng of the recombinant extracellular domain of the partially purified *Bartonella bacilliformis* lipopolysaccharide assembly protein - D (dexr_LptD) (indicated by arrow). Right side: nitrocellulose membrane. B: positive Western blot reaction between dexr_LptD and sera from patients with Carrion’s disease (indicated by arrow).

Finally, the Western blot assay showed a positive reaction between the dexr_LptD protein and sera from patients with CD. For this assay, we used partially purified dexr_LptD and performed electrophoresis of this protein as a control (Figure 5).

## DISCUSSION

The *B. bacilliformis *dexr_LptD protein presented *in silico* linear and discontinuous non-toxic epitopes, and its dominant HLA class I and II binding peptides showed a population coverage of about 100%. At the serological level, dexr_LptD reacted positively with the sera of CD patients during the Western Blot assay.

This study aimed to select a B*. bacilliformis* protein with immunogenic potential that could be used in a future vaccine against CD. Because CD is an emerging infectious disease that is difficult to eradicate with antibiotics or pesticides, the best intervention strategy would be the development of a vaccine based on proteins such as flagellin, Brps (Bartonella repeat proteins), IalB (invasion-associated locus B protein), FtsZ, Hbp/Pap31 (hemon-binding proteins), the α-subunit of succinyl-CoA synthetase (SCS-α), GroEL and the β-subunit of succinyl-CoA synthetase (SCS-β), among others ^4^ ^,^ ^7^ ^,^ ^9^ ^,^ ^10^ ^,^ ^26^. Henríquez-Camacho highlights the importance of studying *B. bacilliformis* membrane proteins in the search for vaccine candidates, since these interact with their host; we used this characteristic as our first filter ^10^.

The bilobular barrel-like structure with the periplasmic N-terminal and C-terminal domain in the outer membrane of LptD from *B. bacilliformis* were reported in the crystal structures of *Yersinia pestis, Klebsiella pneumoniae, Pseudomonas aeruginosa*, *Shigella flexneri* and *Salmonella enterica serovar Typhimurium* ^16^ ^,^ ^17^ ^,^ ^27^. This is evidence that LptD is a conserved protein in gram-negative bacteria. On the other hand, by using the PDB database, we observed high identity of LptD from *B. bacilliformis* with LptD proteins from other species, 30% identity with LptD from *Pseudomonas aeruginosa*, 25% identity with LptD from *Klebsiella pneumoniae*, 28% identity with *Yersinia pestis*, and 21% identity with LptD from *E. coli*, which reinforces the hypothesis that these could be important antigens.

Only the extracellular domain of LptD from *B. bacilliformis* was analyzed because the portion of LptD is accessible to cells of the host immune system; and because the study by Botos *et al*. showed that truncation of the N-terminal portion does not affect the correct folding of the beta-barrel of LptD in different species (*Y. pestis, P. aeruginosa and K. pneumoniae*) when expressed in *E. coli* ^15^. N-terminal truncation of LptD from *B. bacilliformis* was found *in silico* not to affect its normal folding, and the Western Blot assay evidenced the conservation of its epitopes.

On the other hand, LptD proteins from *B. bacilliformis* and LptD from *V. parahaemolyticus* belong to the same protein family (PF04453), therefore, they have similar characteristics and it is expected that LptD from *B. bacilliformis* can induce an immune response in the host as well as its homologue.

Regarding the recombinant expression assays, we observed that the temperature of 28 °C was optimal for induction, while no band was observed at 37 °C, which would be affecting the stability of the induced protein and/or would be accelerating its degradation. 

Usually, membrane proteins are expressed at low levels ^28^ ^,^ ^29^ ^,^ ^30^. In 2015, Chhetri *et al*. proposed a simple and pioneering protocol to enhance recombinant protein expression in *E. coli* with T5 (pQE) or T7 (pET) type expression promoters. Their results showed that adding ethanol at concentrations ≤ 3% (v/v) significantly increased the expression of multiple poorly expressed or unexpressed recombinant proteins ^28^ ^,^ ^29^ ^,^ ^31^. In this study, the dex_lptD gene from *B. bacilliformis* was cloned into a pET 28a (+) plasmid with T7 expression promoter and expressed in *E. coli BL21DE3plySs*, according to the above-mentioned protocol. Given the low expression of the extracellular domain of LptD, we chose to add 3% ethanol to the culture medium at the time of induction with IPTG, which resulted in a significant change in expression. Even with the addition of 1 and 2% ethanol, we observed an increase in expression, with the 3% concentration being optimal, stable and repeatable. More and more studies show the favoring effect of the use of ethanol in the process of recombinant protein expression ^32^, therefore it can be stated that when using ethanol and IPTG in the induction of recombinant LptD from *B. bacilliformis* they achieve a synergistic effect, but ethanol is unable to induce the LptD protein by itself. Zheng *et al*. (2018), reported that ethanol mainly causes an impact on cellular metabolism and cellular stress responses, consequently, the effect of ethanol on heterologous protein expression is very complex ^31^. Cellular stress causes an increase in chaperones, which may favor the folding of heterologous proteins and the impact on cellular metabolism brings about an increase in propionate metabolism and Krebs cycle, this would indirectly increase nutrient input to the cell causing an anabolic effect and favoring heterologous protein expression.

The optimal induction and purification conditions of dexr_LptD from *B. bacilliformis* have allowed us to evaluate its antigenic capacity. Although purification was carried out under denaturing conditions, the recombinant protein was able to react with human sera, which corroborates that LptD possesses multiple linear epitopes that were able to react. As for the discontinuous epitopes, it is very likely that they have lost their 3D structure due to the high concentration of urea (8M) used during the purification process.

The selection criteria for *B. bacilliformis* antigenic proteins and bioinformatics programs used by Dichter *et al*. is similar to our study ^33^. They obtained an antigenic LptD protein that did not show immunoreactivity with sera from people with CD, which contradicts our results. They used the complete protein, whereas we used the extracellular domain of LptD. Another important difference is the amount of recombinant protein used in the western blot assay, they used concentrations of 2.1 ng,10.5 ng and 21 ng, and we used 345 ng of partially purified protein, it is likely that these different concentrations caused different results.

*B. bacilliformis* is a facultative intracellular bacterium, which allows the host to provide a cellular and humoral immune response. The results of the bioinformatic analysis show that LptD has multiple peptides that have strong binding with the most frequent HLA type I and II of the Peruvian population, these peptides should be presented to CD4+ T lymphocytes and CD8+ T lymphocytes which should provoke the activation of the cellular immune response. The positive result of the Western Blot analysis shows that the native LptD protein of *B. bacilliformis* was able to activate the humoral immune response of the host, which produces anti-LptD antibodies that were detected in the serum of people who suffered CD and that were recognized by the dexr_LptD of *B. bacilliformis* in the Western Blot assay. If the immunogenicity of LptD is confirmed, it would be convenient to carry out studies to elaborate a multiepitope protein considering the most immunodominant epitopes of LptD, similarly, Padilla *et al*. proposed a multiepitope protein based on membrane proteins, LptD could be considered within this set of proteins^ (^ ^9^.

The development of a vaccine involves the evaluation of many candidates; however, the use of a bioinformatics selection strategy allows prioritizing the study of few relevant candidates, we focused on the analysis of one protein, expanding the study to more vaccine candidates is necessary. Another limitation is the number of serum samples used in the Western Blot assay, which were a total of five, but this is a preliminary study that could be expanded using a larger number of samples. It should be noted that we carried out the initial part of all the experiments that must be performed in order to develop a vaccine, and these results can be taken into account in future studies.

In conclusion, *in silico* analysis determined that the extracellular domain of the LptD protein of *B. bacilliformis* is antigenic, that it presents epitopes that could be recognized by HLA-I and HLA-II alleles of the Peruvian population, and that it is immunoreactive against human sera from people with CD. This study also highlights the use of 3% ethanol in the induction process of *B. bacilliformis* dexr_LptD. To research further into the topic of the study, we recommend carrying out *in vitro* and *in vivo* assays with the purified dexr_LptD of *B. bacilliformis* purified dexr_LptD, such as *in vitro* assay of the inhibition of erythrocyte invasion by *Bartonella bacilliformis* and immunization of experimental animals (Swiss strain mice and New Zealand breed rabbits) with dexr_LptD followed by inoculation with *Bartonella bacilliformis* in order to evaluate the immunoprotective effect of the recombinant protein.
