# Supplementary material for: Cloning, expression and seroreactivity of the recombinant lipopolysaccharide assembly protein - D (LptD) from Bartonella bacilliformis
Source: Rev Peru Med Exp Salud Publica. 2022 Mar 31;39(1):15–23. doi: 10.17843/rpmesp.2022.391.9292 (PMC11397601; doi:10.17843/rpmesp.2022.391.9292)
Supplement: Supplementary material. — Available in the electronic version of the RPMESP. [file rpmesp-39-01-9292-s001.pdf]

MATERIAL SUPLEMENTARIO

Anexo 1. HLA-I y HLA-II más frecuentes en la población peruana

Predicción de unión a HLA-I-9aa		Predicción de unión a HLA-II-11aa	
**HLA más frecuentes**	**HLA menos frecuentes pero importantes**	**HLA más frecuentes**	**HLA menos frecuentes pero importantes**
HLA-A*02:01	HLA-A*01:01		
HLA-A*23:01	HLA-A*03:01		
HLA-A*24:02	HLA-A*26:01		
HLA-A*31:01	HLA-A*30:01		HLA-DRB1*01:01
HLA-A*68:01	HLA-A*33:01	HLA-DRB1*04:01	HLA-DRB1*03:01
HLA-B*07:02	HLA-B*08:01	HLA-DRB1*08:01	HLA-DRB1*07:01
HLA-B*15:01	HLA-B*14:01	HLA-DRB1*09:01	HLA-DRB1*11:01
HLA-B*35:01	HLA-B*18:01	HLA-DRB1*13:01	HLA-DRB1*15:01
HLA-B*39:01	HLA-B*27:01		HLA-DRB1*16:02
HLA-B*40:01	HLA-B*38:01		
HLA-B*44:02	HLA-B*49:01		
HLA-B*48:01	HLA-B*52:01		
HLA-B*51:01			

Fuente: Comunicación personal del Blgo. Adolfo Marcelo – Laboratorio de Referencia Nacional de Metaxénicas Virales, Centro Nacional de Salud Pública, Instituto Nacional de Salud. Lima, Perú.

Rev Peru Med Exp Salud Publica
https://doi.org/10.17843/rpmesp.2022.381.9292
