# Supplementary material for: Cloning, expression and seroreactivity of the recombinant lipopolysaccharide assembly protein - D (LptD) from Bartonella bacilliformis
Source: Rev Peru Med Exp Salud Publica. 2022 Mar 31;39(1):15–23. doi: 10.17843/rpmesp.2022.391.9292 (PMC11397601; doi:10.17843/rpmesp.2022.391.9292)
Supplement: Translation

# Translation: Clonamiento, expresión y seroreactividad de la proteína recombinante de ensamblaje de lipopolisacáridos - D (LptD) de *Bartonella bacilliformis*

**Authors:** Astrid Flores-Nuñez, Gladis Ventura, Henri Bailon, Adolfo Marcelo, Gustavo Sandoval, Carlos Padilla-Rojas

**Affiliations:** 1 Laboratorio de Biotecnología y Biología Molecular, Centro Nacional de Salud Pública, Instituto Nacional de Salud, Lima, Perú.; 2 Grupo de Investigación en Bioinformática y Biología Estructural, Universidad Nacional Mayor de San Marcos, Lima, Perú.; 3 Laboratorio de Referencia Nacional de Metaxénicas Virales, Centro Nacional de Salud Pública, Instituto Nacional de Salud, Lima, Perú.

**Keywords:** Infecciones por *Bartonella*, proteína LptD, *Bartonella bacilliformis*, Proteínas recombinantes, Antigenicidad vacunal, Biología computacional

## Abstract

**Objetivo.:**

Evaluar *in silico *y a nivel serológico el potencial antigénico del dominio extracelular recombinante de la proteína de ensamblaje de lipopolisacáridos - D (LptD) de *Bartonella bacilliformis *(dexr_LptD).

**Materiales y métodos.:**

Mediante el análisis *in silico* se realizó la selección de una proteína de *B. bacilliformis *con potencial antigénico e inmunogénico. El gen de la proteína seleccionada se clonó en *Escherichia coli TOP10* y se expresó en *Escherichia coli BL21 (DE3) pLysS. *La proteína recombinante fue expresada usando isopropil-β-D-1-tiogalactopiranósido (IPTG) y se optimizaron las condiciones de inducción. Por último, se purificó con resina Ni-IDA (His60 Ni *Superflow*) y se realizó un ensayo de Western Blot.

**Resultados.:**

*In silico,* la proteína seleccionada fue LptD por estar localizada en la membrana externa y ser antigénica e inmunogénica. Las condiciones optimizadas para la inducción del dexr_LptD fueron 0,5 mM IPTG, 16 h, medio TB (Terrific Broth), etanol al 3% (v/v), 28 ºC, OD600: 1-1,5 y 200 r.p.m. La purificación se realizó en condiciones denaturantes a pequeña escala y se obtuvo 2,6 µg/mL de dexr_LptD parcialmente purificada. El ensayo de Western Blot mostró una reacción positiva entre los sueros provenientes de pacientes con la enfermedad de Carrión y dexr_LptD, ello evidencia la antigenicidad del dexr_LptD.

**Conclusiones.:**

El dexr_LptD muestra antigenicidad *in silico* y a nivel serológico, estos resultados son base para posteriores estudios sobre candidatos vacunales contra la enfermedad de Carrión.

## INTRODUCCIÓN

*Bartonella bacilliformis* es el agente etiológico de la enfermedad de Carrión (EC), cuyo vector es el flebótomo del género *Lutzomyia spp.* La EC es una enfermedad desatendida en Perú, Colombia y Ecuador y en la primera fase de su desarrollo tiene un alto índice de letalidad y mortalidad (40 - 88%) en pacientes sin tratamiento antimicrobiano ^1^. Las investigaciones con relación a la EC se han direccionado a la indagación de técnicas de detección más sensibles, como la reacción en cadena de la polimerasa (PCR) ^2^; a la identificación de nuevas proteínas antigénicas e inmunogénicas para ser usadas en el diagnóstico serológico ^3^ ^-^ ^8^ y muy pocas en la búsqueda de candidatos vacunales ^9^ ^,^ ^10^, por lo que, en la actualidad, no se cuenta con una vacuna para esta enfermedad.

La vacunología reversa, metodología utilizada en el presente estudio, nos permite la identificación de antígenos e inmunógenos sin la necesidad de cultivar al patógeno; esto lo logra a través del uso de programas bioinformáticos, paso previo a los ensayos experimentales ^11^. Esta metodología permitió sintetizar una proteína multiepitópica de *B. bacilliformis, *la cual, logró inducir una respuesta inmune en ratones, lo que la posiciona como un buen candidato vacunal contra la EC ^9^.

Las proteínas de membrana externa son las indicadas dentro del proteoma de un microorganismo para la búsqueda de candidatos vacunales ^12^ ^,^ ^13^, debido a su capacidad de interactuar con el sistema inmunológico del hospedero. Así tenemos, al sistema de transporte de lipopolisacáridos (LPS) LptABCDEFG, un complejo proteico, responsable de la translocación de los LPS desde la membrana interna hacia la membrana externa^ (^ ^14^ ^-^ ^16^. La proteína de ensamblaje de lipopolisacáridos - D (LptD), parte del sistema LptABCDEFG, ha mostrado ser altamente inmunogénica, en consecuencia, un buen candidato vacunal en otros microorganismos como *Vibrio parahaemolyticus*; además, podría a ser utilizada en inmunoterapia por ser pieza clave en el proceso final de translocación de LPS ^17^ ^,^ ^18^.

Ante lo expuesto, la presente investigación tiene como objetivo evaluar *in silico* y a nivel serológico el potencial antigénico del dominio extracelular recombinante de la proteína de ensamblaje de lipopolisacáridos - D (LptD) de *Bartonella bacilliformis *(dexr_LptD). Los resultados obtenidos serán de importancia en la línea de investigación básica para el desarrollo de una vacuna contra la EC.

**MENSAJES CLAVE**

Motivación para realizar el estudio: actualmente no se cuenta con una vacuna contra la enfermedad de Carrión, la cual afecta a poblaciones vulnerables. Por otro lado, tenemos a la vacunología reversa como una herramienta que nos ayuda en la selección de candidatos vacunales.

Principales hallazgos: el dominio extracelular recombinante de la proteína de ensamblaje de lipopolisacáridos - D (LptD) de *B. bacilliformis* mostró antigenicidad* in silico* y a nivel serológico.

Implicancias: los resultados contribuyen en la línea de investigación sobre candidatos vacunales contra la enfermedad de Carrión.

## MATERIALES Y MÉTODOS

La metodología presentó dos etapas: el análisis *in silico* y los ensayos experimentales (Figura 1).

**Figura 1:** Flujograma de trabajo. Análisis* in silico*: muestra los programas bioinformáticos y criterios para la selección de la proteína en estudio. Ensayos experimentales: secuencia de procedimientos realizados para la obtención y evaluación dominio extracelular recombinante de la proteína de ensamblaje de lipopolisacáridos - D de *Bartonella bacilliformis* (dexr_LptD). LptD (proteína de ensamblaje de lipopolisacáridos - D), HLA-I y HLA-II (antígenos leucocitarios humanos I y II), IPTG (isopropil-β-D-tiogalactopiranósido). Programas bioinformáticos: SignalP4., VaxiJen v2.0 y Vaxign, ProtParam/ Raptor X Structure Prediction/ plot de Ramachandran, Ellipro, Toxin Pred, NetMHCpan 4.0 Server y NetMHCII 2.3 Server, Servidor IEDB, PrimerSelect 4.05 y OligoAnalyzer 3.1.

### Análisis *in silico*

A partir del genoma y proteoma de *B. bacilliformis* KC583 (ID Assembly: GCA_000015445.1) se seleccionó una proteína de membrana externa con potencial antigénico e inmunogénico. El programa SignalP4.1 (https://services.healthtech.dtu.dk/service.php?SignalP-4.1) fue el primer filtro, este permitió seleccionar a las proteínas con péptido señal, es decir, proteínas ubicadas en la membrana celular. Signal P4.1 es uno de los mejores predictores de péptido señal ^19^. Los siguientes filtros se realizaron con los programas VaxiJen v2.0 (http://www.ddg-pharmfac.net/vaxijen/VaxiJen/VaxiJen.html) y Vaxign (http://www.violinet.org/vaxign/), los cuales permitieron seleccionar a las proteínas ubicadas en la membrana externa con potencial antigénico e identificar la similitud con las proteínas humanas, de ratón o de cerdo, así como estimar la longitud de la proteína y su probabilidad de ser adhesinas. VaxiJen v2.0 y Vaxign son programas con interfaz gráfica rápida, uso intuitivo, que se actualizan con regularidad y han sido los más citados en comparación con los programas Nerve, Jenner-predict, Bowman-Heinson y VacSol ^20^ ^,^ ^21^.

Las propiedades fisicoquímicas (peso molecular, el pI teórico, la composición de aminoácidos, la composición atómica, el coeficiente de extinción, la vida media estimada, el índice de inestabilidad y el índice alifático) de las proteínas preseleccionadas se obtuvieron con ProtParam (https://web.expasy.org/protparam/). La modelización de las proteínas fue con RaptorX Property (http://raptorx2.uchicago.edu/StructurePropertyPred/predict/) y se validaron con el plot de Ramachandran, a través del programa Rampage: Ramachandran Plot Assessment (http://mordred.bioc.cam.ac.uk/~rapper/rampage.ph). Cabe indicar que RaptorX Property se encuentra entre los cinco mejores programas automatizados de modelamiento de proteínas ^22^. La orientación espacial de la proteína se evaluó con el programa PPM server (https://opm.phar.umich.edu/ppm_server).

Una vez obtenida la proteína de interés, debía identificarse la presencia de epítopes lineales y discontinuos, para ello se utilizó el servidor Ellipro (http://tools.iedb.org/ellipro/). Este servidor selecciona los péptidos con un score mayor a 0,5 y los califica como antígenos. Por otra parte, Toxin Pred - Protein Scanning (http://crdd.osdd.net/raghava/toxinpred/) evaluó la toxicidad de los péptidos.

Una proteína con potencial vacunal debe poseer epítotes capaces de interactuar con el sistema del antígeno leucocitario humano (HLA) de clase I y II, la identificación de péptidos con unión fuerte a HLA-I y II se realizó con los programas NetMHCpan 4.0 Server (https://services.healthtech.dtu.dk/service.php?NetMHCpan-4.1) y NetMHCII 2.3 Server (https://services.healthtech.dtu.dk/service.php?NetMHCII-2.3), respectivamente.

El programa IEDB (https://www.iedb.org/) evaluó el porcentaje de cobertura, a nivel nacional, sudamericano y mundial, de la interacción de los HLA-I y HLA-II más frecuentes de la población peruana con los péptidos seleccionados. En el Anexo 1 se muestra los HLA-I y HLA-II más frecuentes e importantes en la población peruana (datos obtenidos por comunicación con el Blgo. Adolfo Marcelo del Instituto Nacional de Salud de Perú).

## ENSAYOS EXPERIMENTALES

### Diseño de oligonucleótidos

Los oligonucleótidos se diseñaron con los programas PrimerSelect 4.05 (ADNSTAR Inc. Madison, USA) y OligoAnalyzer 3.1 de IDT (www.idtdna.com). Los oligonucleótidos *forward* y *reverse* fueron modificados para incluir los sitios de cortes para las enzimas de restricción *Nco*I (C/CATGG) y *Xho*I (C/TCGAG), respectivamente. Una cola de seis Timinas fue añadida en el extremo 5’ de los oligonucleótidos para asegurar la eficiencia del corte. Los oligonucleótidos fueron: *forward* 5΄-TTTTTTCCATGGTACCGGACCCAGCTGAAA-3΄, y *reverse* 5΄-TTTTTTCTCGAGTAAATTGAGTTTTATTTTTTGACCGAAATCT-3΄.

### Amplificación del gen del dominio extracelular de lptD (gen *dex_lptD*)

Para la amplificación del gen mediante PCR ^23^ se usó el ADN genómico de *B. bacilliformis cepa HBB4* de Huacabamba (Piura, Perú). El kit utilizado fue el AmpliTaq Gold™ ADN Polymerase con Gold buffer y MgCl_2_ (Applied Biosystems). Las concentraciones del *buffer* Mix de PCR fueron: MgCl_2_ 2.5 mM, *Gold buffer* 1X, 1 µM de oligonucleótidos *forward*, 1 µM de oligonucleótidos *reverse*, 0.2 mM de dNTPs y 1 U/reacción de Taq Pol. El ciclaje del PCR consistió en una desnaturalización inicial a 95 ºC por 10 min, seguido de 35 ciclos de desnaturalización a 96 ºC por 30 s, hibridación a 58 °C por 30 s y amplificación a 72 ºC por 2 min; por último, una amplificación a 72 ºC por 10 min.

### Clonación del gen *dex_lptD* en *E. coli*

El gen *dex_lptD* amplificado y el plásmido de expresión pET28a(+) (Promega Corp.,USA) fueron digeridos con las enzimas de restricción *Nco*I y *Xho*I, y purificados con el kit PureLink™ Quick Plasmid Miniprep (Invitrogen Inc, USA). Luego se procedió a su ligación con T4 ligasa (Promega Corp.,USA) y se formó el plásmido pET28a (+) - *dex_lptD.*

Las cepas *E. coli TOP10* (cepa de clonación) y *E. coli Bl21 (DE3) pLysS* (cepa de expresión), competentes, fueron transformadas mediante el método de cloruro de calcio ^24^. La primera transformación se dio entre *E. coli TOP 10 *y el plásmido pET28a (+) - *dex_lptD, *ello result*ó* en* E. coli TOP 10 - dex_lptD*, de esta se extrajeron los plásmidos según el método de lisis alcalina ^25^. Los plásmidos extraídos sirvieron para realizar la segunda transformación y se obtuvo *E. coli BL21(DE3)pLysS - dex_lptD*.

Con la finalidad de comprobar la conservación de la secuencia del gen *dex_lptD* en las clonas transformadas se secuenciaron según el método de Sanger *et al.* (1977) y Murray (1989), para ello se utilizó el kit Big Dye Terminator v3.1 Cycle Sequencing (AppliedBiosystems), el secuenciador ABI, y el programa Chromas Pro para su respectivo análisis.

### Expresión y purificación de la proteína dexr_LptD

La expresión de la proteína recombinante se optimizó evaluando cuatro parámetros: (a) concentración de IPTG (Sigma-Aldrich Merck, USA): 0, 0.05, 0.1, 0.25, 0.5, 1, 1.5, 2 mM IPTG; (b) medios de cultivo: LB (Luria Bertani), 2XYT (Yeast Extract Tryptone) y TB (Terrific Broth); (c) tiempo de inducción: 4, 7, 16 y 24 horas; (d) concentración de etanol: 1%, 2 % y 3% e) inducción a diferentes temperaturas 20 oC, 28 oC y 37 oC. Los medios de cultivo fueron suplementados con kanamicina (20 µg/mL) y con cloranfenicol (50 µg/mL).

Para la purificación se evaluó dos condiciones: denaturantes (8M úrea) y no denaturantes. La purificación fue por afinidad con 50 µL de resina His60 Ni *Superflow* (Clontech) y se siguió las recomendaciones del fabricante. La cuantificación de proteínas se realizó con el método de Bradford.

### Electroforesis PAGE-SDS y detección por Western Blot

La electroforesis PAGE-SDS de la proteína parcialmente purificada se realizó en un gel de poliacrilamida al 12%, se fotografió el gel y se analizó usando el programa QuantityOne 4.6.9 1-D Analysis.

El ensayo de Western Blot se realizó en una cámara de transferencia semiseco horizontal (3 mA/ cm^2^ por 30 min) y se utilizó una mezcla diluida de cinco sueros humanos (0,1%). Estos sueros procedentes de la seroteca del Laboratorio Biotecnología y Biología Molecular (INS) pertenecían a personas que padecieron la EC. Para este ensayo se utilizó 345 ng de proteína recombinante parcialmente purificada. Por último, se fotografió la membrana y fue analizada con el programa QuantityOne 4.6.9 1-D *Analysis Software*.

## RESULTADOS

El análisis *in silico* del proteoma de *B. bacilliformis *KC583 nos permitió seleccionar la proteína LptD (ID: A1US68_BARBK) por cumplir con los criterios de selección: ser antigénica (0,562), localización en la membrana externa, no presentar similitud con proteínas humanas, de ratón, o de cerdo, y la longitud de la proteína fue mayor a 10 kDa (86,80 KDa). Los posteriores ensayos se realizaron solo con el dominio extracelular de LptD (67,42 kDa, pI 7,86) por ser la porción de la proteína LptD que se ubica hacia el medio extracelular, por ende, con probabilidad de interactuar con el sistema inmune del hospedero. El dexr_LptD forma un canal y posee como aminoácidos más abundantes a la asparagina (8,9%) y a la serina (8,4%) (Figura 2). El programa RaptorX logró modelar el 100% de la secuencia de aminoácidos del dexr_LptD, esta utilizó como plantillas a proteínas homólogas de LptD de diferentes especies: *E. coli* (PDB: 4RHBA), *Pseudomonas aeruginosa* (PDB: 5IVA), *Yersinia pestis* (5IXMA) y *Shigella flexneri* (PDB: 4Q35A). Así mismo, este dominio presentó 18 epítopes lineales y cinco epítopes discontinuos; además, ninguno de los 569 péptidos evaluados resultó ser tóxico. Se obtuvo una alta cobertura poblacional de los péptidos dominantes de unión a HLA clase I y clase II, estos fueron de 100,00% para el Perú, 93,38% para Sudamérica y 99,28% a nivel mundial.

**Figura 2:** Modelo 3D del dominio extracelular recombinante de ensamblaje de lipopolisacáridos - D de *Bartonella bacilliformis* (dexr_LptD). A: vista lateral, B: vista superior. Estructuras secundarias: láminas beta (de color amarillo), hélice alfa (de color rojo), loops (de color verde) y la cola de histidina (de color blanco).

En la transformación de las cepas *E. coli TOP10* se obtuvieron 116 colonias en la placa de ligado y cuatro colonias en la placa de autoligado, lo que indica que la mayoría de las bacterias transformadas presentaron plásmido pET28a (+) - *dex_lptD*. Se realizó un PCR colonia a seis colonias seleccionadas al azar de la placa de ligado y todas mostraron productos de amplificación en el tamaño esperado (1756 pb). Se secuenció el gen *lptD* de *E. coli TOP10 *- *dex_lptD* y no se observó cambios nucleotídicos respecto al molde (gen *dex_lptD* de *B. bacilliformis* KC583). Una vez confirmadas la secuencia del gen *dex_lptD* mediante secuenciación se procedió a realizar la transformación en la cepa de expresión *E. coli BL21 (DE3) pLysS, *y se escogió cuatro colonias al azar para realizar PCR colonia. Los resultados de PCR confirmaron la correcta transformación en *E. coli BL21(DE3)pLysS*.

Las condiciones óptimas de inducción del dexr_LptD fueron: 0,5 mM IPTG, medio TB, 16 horas, 3% de etanol, 28 ºC y 200 rpm. La purificación optimizada del dexr_LptD contó con dos pasos de lisis (la primera no denaturante y la segunda denaturante), ya que, al realizarse únicamente con *buffer* denaturante se obtuvo una gran cantidad de bandas inespecíficas en la electroforesis (Figura 3). La importancia del primer paso de la lisis recae en la eliminación de las proteínas nativas existentes en la muestra que interactuaban con la resina de purificación. Cabe indicar que en condiciones no denaturantes no se logró purificar la proteína (Figura 4). La cantidad de proteína parcialmente purificada en condiciones óptimas cuantificadas mediante el método de Bradford fue de 2,6 µg/mL del dexr_LptD.

**Figura 3:** Proceso de optimización de la expresión del dominio extracelular recombinante de ensamblaje de lipopolisacáridos - D de *Bartonella bacilliformis* (dexr_LptD), donde M (marcador: Unstained Protein Standards) y las flechas indican la altura del dexr_LptD (67,42 kDa) (banda inducida).

**Figura 4:** Purificación parcial del dominio extracelular recombinante de la proteína de ensamblaje de lipopolisacáridos - D de *Bartonella bacilliformis* (dexr_LptD) en condiciones denaturantes y no denaturantes. M: Marcador: Unstained Protein Standards. D: purifación parcial bajo condiciones denaturantes. ND: purificación parcial bajo condiciones no denaturantes. Las flechas indican la altura del dexr_LptD (67,42 kDa).

Finalmente, el ensayo de Western Blot mostró una reacción positiva entre la proteína dexr_LptD y los sueros de pacientes que sufrieron la EC, para este ensayo se utilizó el dexr_LptD parcialmente purificada, se realizó a la par una electroforesis de ésta misma a modo de control (Figura 5).

**Figura 5:** Western Blot. M: Marcador: Prestained Natural SDS-PAGE *Protein Standards*. Lado izquierdo: Gel de PAGE-SDS. A: 115 ng del dominio extracelular recombinante de la proteína de ensamblaje de lipopolisacáridos - D de *Bartonella bacilliformis* (dexr_LptD) parcialmente purificada (indicado con la flecha). Lado derecho: membrana de nitrocelulosa. B: reacción positiva del Western Blot realizado entre dexr_LptD y sueros de pacientes que padecieron la enfermedad de Carrión (indicado con la flecha).

## DISCUSIÓN

La proteína dexr_LptD de *B. bacilliformis* presentó *in silico* epítopes no tóxicos lineales y discontinuos, además resaltó que sus péptidos dominantes de unión a HLA clase I y II presentaron una cobertura poblacional alrededor del 100%. A nivel serológico, dexr_LptD logró una reacción positiva con los sueros de los pacientes que sufrieron la EC en el ensayo de Western Blot.

El presente trabajo tuvo como objetivo seleccionar una proteína de *B. bacilliformis* con potencial inmunogénico que pueda ser usado en una futura vacuna contra la EC. Debido a que la EC es una enfermedad infecciosa emergente difícil de erradicar con antibióticos o pesticidas, la mejor estrategia de intervención sería el desarrollo de una vacuna basada en proteínas como flagelina, Brps (*Bartonella repeat proteins*), IalB (*invasion-associated locus B protein*), FtsZ, Hbp/Pap31 (*hemon-binding proteins*), la subunidad α de succinil-CoA sintetasa (SCS-α), GroEL y la subunidad β de succinil-CoA sintetasa (SCS-β), entre otras ^4^ ^,^ ^7^ ^,^ ^9^ ^,^ ^10^ ^,^ ^26^. Henríquez-Camacho resalta la importancia del estudio de proteínas de membranas de *B. bacilliformis* en la búsqueda de candidatos vacunales, ya que estas interactúan con su hospedero, en nuestra selección dicha característica fue nuestro primer filtro ^10^.

La estructura tipo barril bilobular con el dominio N-terminal periplasmático y C-terminal en la membrana externa de LptD de *B. bacilliformis*, fueron reportadas en las estructuras cristalinas de *Yersinia pestis, Klebsiella pneumoniae, Pseudomonas aeruginosa, Shigella flexneri *y* Salmonella enterica serovar Typhimurium* ^16^ ^,^ ^17^ ^,^ ^27^. Esto evidencia que LptD es una proteína conservada en bacterias gramnegativas. Por otro lado, empleando la base de datos PDB se observó una alta identidad de LptD de *B. bacilliformis* con proteínas LptD de otras especies, así tenemos un 30% de identidad con LptD de *Pseudomonas aeruginosa*, 25% de identidad con LptD de *Klebsiella pneumoniae*, 28% de identidad con *Yersinia pestis*, y 21% de identidad con LptD de E*. coli*, lo cual refuerza la hipótesis que se tratarían de antígenos importantes.

Se analizó solo el dominio extracelular de LptD de *B. bacilliformis* debido a que la porción de LptD que es accesible a las células del sistema inmune del huésped; y porque el trabajo de Botos *et al. *evidenció que el truncamiento de la porción N terminal no afecta al correcto plegamiento del barril beta de LptD en diferentes especies (*Y. pestis, P. aeruginosa y K. pneumoniae*) cuando son expresadas en *E. coli* ^15^. *In silico *se demostró que el truncamiento N terminal de LptD de *B. bacilliformis* no afectó su normal plegamiento, y en el ensayo Western Blot evidenció la conservación de sus epítopes.

Por otro lado, las proteínas LptD de *B. bacilliformis* y LptD de *V. parahaemolyticus* pertenecen a la misma familia de proteínas (PF04453), por ende, presentan características comunes y se espera que LptD* de B. bacilliformis* pueda inducir una respuesta inmune en el huésped al igual que lo hace su homólogo.

Con respecto a los ensayos de expresión recombinante, se observó que la temperatura de 28 °C fue óptima para la inducción, mientras que a 37 °C no se presentó ninguna banda, la cual estaría afectando la estabilidad de la proteína inducida y/o estaría acelerando su degradación.

Generalmente, las proteínas de membrana se expresan en niveles bajos ^28^ ^,^ ^29^ ^,^ ^30^. En el 2015, Chhetri *et al.* propusieron un protocolo simple y pionero para mejorar la expresión de proteínas recombinantes en *E. coli *con promotores de expresión del tipo T5 (pQE) o T7 (pET). Sus resultados mostraron que agregando etanol a concentraciones ≤ 3% (v/v) se logra aumentar significativamente la expresión de múltiples proteínas recombinantes poco expresadas o no expresadas ^28^ ^,^ ^29^ ^,^ ^31^. En el presente trabajo, el gen *dex*_*lptD* de *B. bacilliformis* fue clonado en un plásmido pET 28a (+) con promotor de expresión T7 y expresado en *E. coli BL21DE3plySs*, de acuerdo con el protocolo antes mencionado. Ante la baja expresión del dominio extracelular de LptD se optó por agregar 3% de etanol al medio de cultivo en el momento de la inducción con IPTG, con lo cual se logró un cambio significativo en la expresión. Inclusive agregando etanol al 1 y 2% se observó aumento en la expresión, siendo la concentración de 3 % óptima, estable y repetitiva. Cada vez más trabajos muestran el efecto favorecedor del uso de etanol en el proceso de expresión de proteínas recombinantes ^32^, por ello se puede afirmar que al utilizar etanol e IPTG en la inducción de LptD recombinante de *B. bacilliformis* logran un efecto sinérgico, pero el etanol es incapaz de inducir la proteína LptD por sí solo. Zheng *et al.* (2018), afirman que el etanol causa principalmente un impacto en el metabolismo celular y respuestas a estrés celular, en consecuencia, el efecto del etanol en la expresión de proteínas heterólogas es muy complejo ^31^. El estrés celular provoca un incremento de chaperonas, lo cual puede favorecer al plegamiento de las proteínas heterólogas y el impacto en el metabolismo celular trae consigo el aumento del metabolismo del propanoato y del ciclo de Krebs, ello indirectamente incrementaría la entrada de nutrientes a la célula provocando un efecto anabólico y favoreciendo la expresión de proteínas heterólogas.

Las condiciones óptimas de inducción y purificación del dexr_LptD de *B. bacilliformis* han permitido evaluar su capacidad antigénica. A pesar de que la purificación fue en condiciones denaturantes, la proteína recombinante fue capaz de reaccionar con los sueros humanos, ello corrobora que LptD posee múltiples epítopes lineales que fueron capaces de reaccionar. En cuanto a los epítopes discontinuos, es muy probable hayan perdido su estructura 3D por la alta concentración de urea (8M) usada en el proceso de purificación.

Dichter* et al.* utilizaron criterios de selección de las proteínas antigénicas de *B. bacilliformis *y programas bioinformáticos similares a nuestro estudio ^33^. Ellos obtuvieron una proteína LptD antigénica que no mostró inmunorreactividad con sueros procedentes de personas con EC, lo que es contrario a nuestros resultados. Ellos utilizaon la proteína completa, mientras que nosotros usamos el dominio extracelular de LptD. Otra diferencia importante es la cantidad de proteína recombinante utilizada en el ensayo de Western Blot, ellos utilizaron concentraciones de 2,1 ng,10,5 ng y 21 ng, y nosotros usamos 345 ng de proteína parcialmente purificada, es probable que estas diferencias en las concentraciones produjeran resultados distintos.

*B. bacilliformis* es una bacteria intracelular facultativa, ello permite al hospedero dar una respuesta inmune celular y humoral. Los resultados del análisis bioinformático nos muestran que LptD presenta múltiples péptidos que poseen unión fuerte con los HLA tipo I y II más frecuentes de la población peruana, dichos péptidos deben ser presentados a los linfocitos T CD4+ y linfocitos T CD8+ y provocar la activación de la respuesta inmune celular. El resultado positivo del análisis por Western Blot, muestra que la proteína LptD nativa de *B. bacilliformis* fue capaz de activar la respuesta inmunitaria humoral del huésped, la cual produce anticuerpos anti-LptD que fueron detectados en el suero de personas que sufrieron la EC y reconocidos por el dexr_LptD de *B. bacilliformis* en el ensayo de Western Blot. De comprobarse la inmunogenicidad de LptD sería conveniente realizar estudios de elaboración de una proteína multiepitópica considerando los epítopos más inmunodominantes de LptD, así como, Padilla *et al.* proponen una proteína multiepitópica en base a proteínas de membrana, podría LptD ser considerada dentro de este conjunto de proteínas ^9^.

La elaboración de una vacuna implica la evaluación de muchos candidatos, sin embargo, el uso de una estrategia de selección bioinformática permite priorizar el estudio de pocos candidatos relevantes, nosotros nos enfocamos en el análisis de una proteína, es deseable ampliar el estudio a más candidatos vacunales. Otra limitante que podemos mencionar es el número de muestras de suero utilizado en el ensayo de Western Blot, que fueron un total de cinco, pero este es un estudio preliminar que podría ser ampliado utilizando un mayor número de muestras. Cabe resaltar que nosotros llevamos a cabo la parte inicial de todos los experimentos que se deben realizar para poder desarrollar una vacuna, y estos resultados pueden ser tomados en cuenta en futuros trabajos.

En conclusión, el análisis *in silico* determinó que el dominio extracelular de la proteína LptD de *B. bacilliformis* es antigénica, que presenta epítopes que podrían ser reconocidos por alelos de HLA-I y HLA-II de la población peruana y que es inmunorreactivo contra sueros humanos de personas con EC. El presente estudio además resalta el uso del etanol al 3% en el proceso de inducción del dexr_LptD de *B. bacilliformis*. Con el objetivo de ahondar el estudio se recomienda realizar ensayos *in vitro* e *in vivo* con el dexr_LptD de *B. bacilliformis* purificada tales como, el ensayo *in vitro* de la inhibición de la invasión de eritrocitos por *Bartonella bacilliformis* e inmunizaciones a animales de experimentación (ratones de cepa Swiss y conejos de la raza New Zealand) con dexr_LptD seguido de una inoculación con *Bartonella bacilliformis* con el fin de evaluar el efecto inmunoprotector de la proteína recombinante.
